# Algernon Thomas Bristow, M. D.

**Published:** 1913-05

**Authors:** 


Algernon Thomas Bristow, M. D., College of Physicians and Surgeons, New York City, 1876; a member of the American Medical Association; American Academy of Medicine, and American Surgical Association; once vice-president of the New York Academy of Medicine; editor of the *New York State Journal of Medicine;* clinical professor of surgery in the Long Island College Hospital, Brooklyn; consulting surgeon of St. John's Long Island College, Long Island State, Swedish Coney Island and Bushwick Hospitals; representative of the Medical Society of the State of New York in the House of Delegates of the American Medical Association continuously since 1906 except at the 1908 annual session; chairman of the committee of the American Medical Association to inquire of the British Medical Association whether or not a joint meeting of these two organizations is desirable and possible; a prolific writer, and always an active worker for the betterment of the profession and higher ideals in medicine and surgery, died at his home in Brooklyn, March 26, aged 61, from septicemia, the result of a prick of the finger while operating two weeks before. (*Jour. of the A. M. A.*) We take this opportunity to remind our readers that the great majority of medical editors, especially in America, are engaged in active practice and are subject to the same risks and demands upon their time as other practitioners. While this fact sometimes results in minor imperfections in the preparations of journals, it has the great advantage that the editorial point of view of matters of public interest is much more apt to represent the genuine needs of the profession and the editorial judgment in the selection of technical articles is much more practical and sound, than if the editors were merely office men.

